# Supplementary figures and images for: Cooperative effects of RIG-I-like receptor signaling and IRF1 on DNA damage-induced cell death
Source: Cell Death Dis. 2022 Apr 18;13(4):364. doi: 10.1038/s41419-022-04797-7 (PMC9016077; doi:10.1038/s41419-022-04797-7)

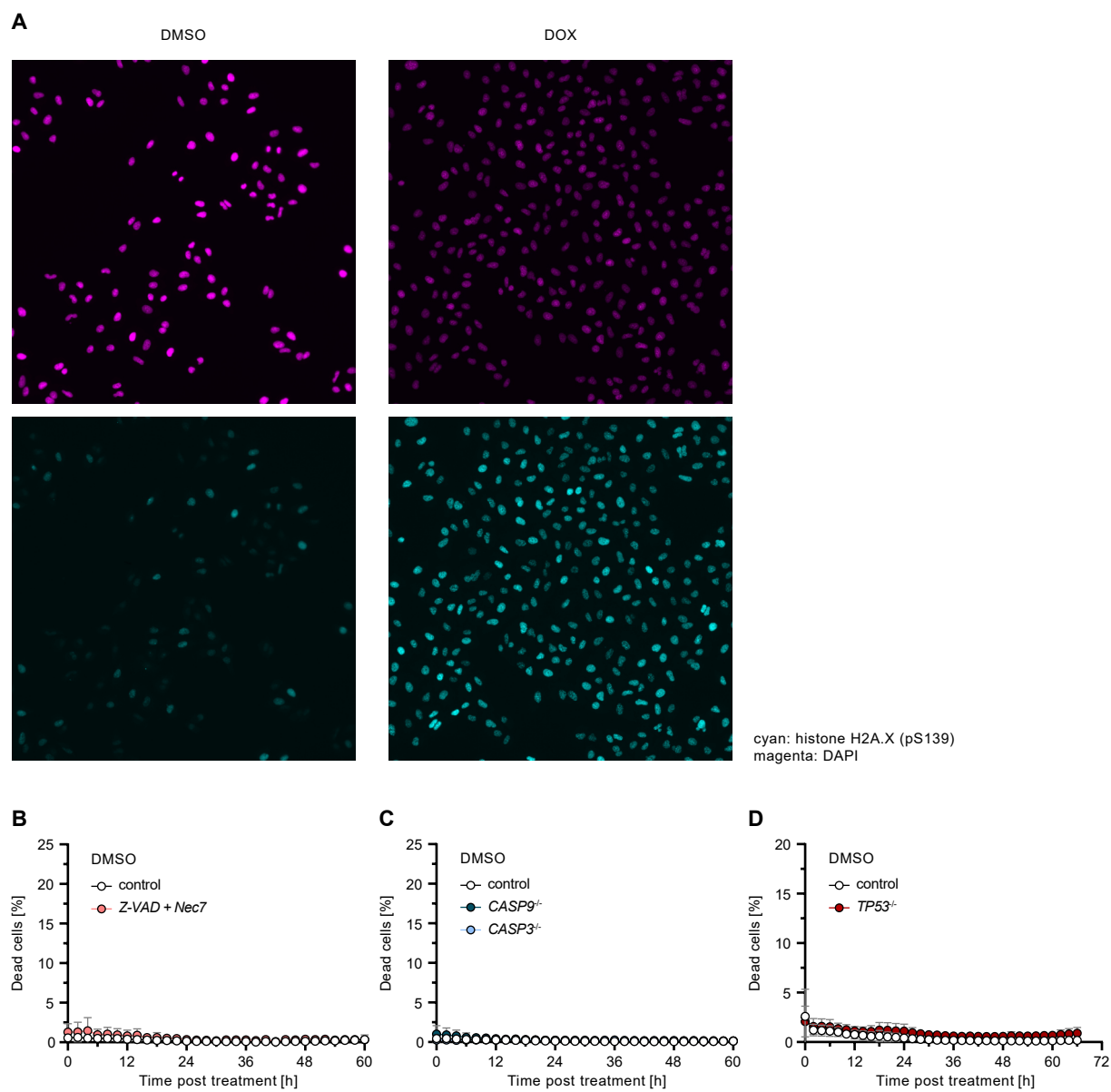

**Fig. S1**

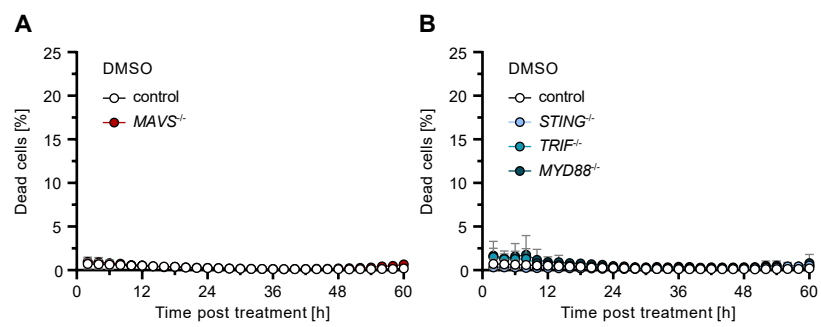

**Fig. S2**

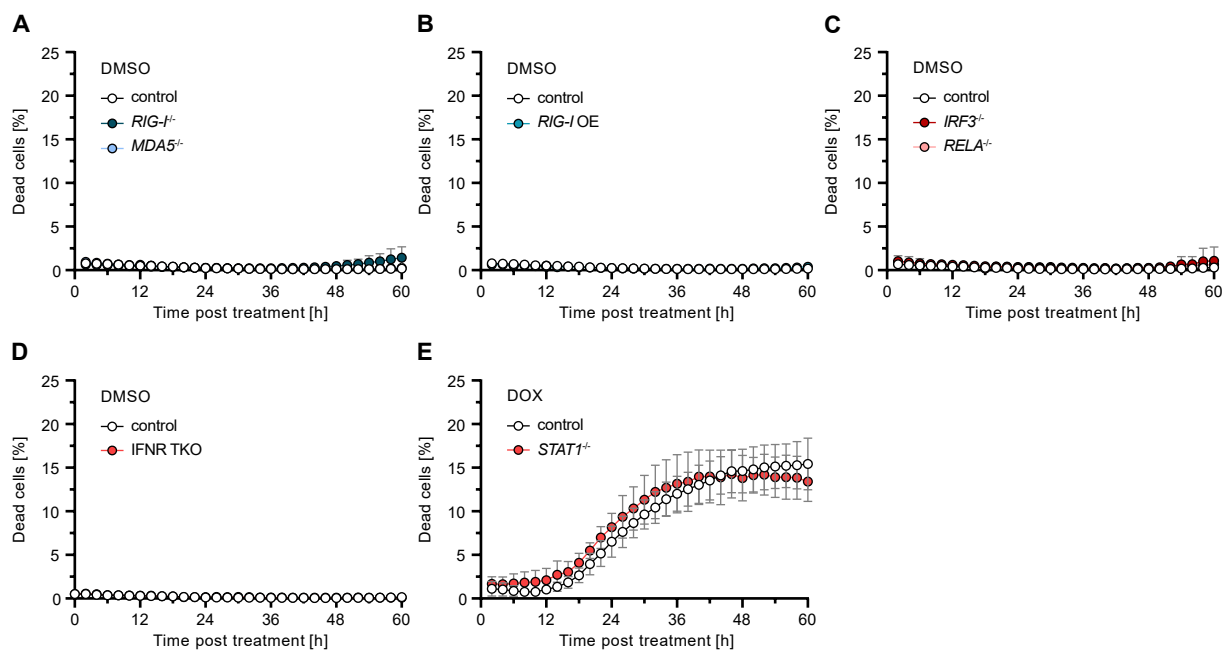

**Fig. S3**

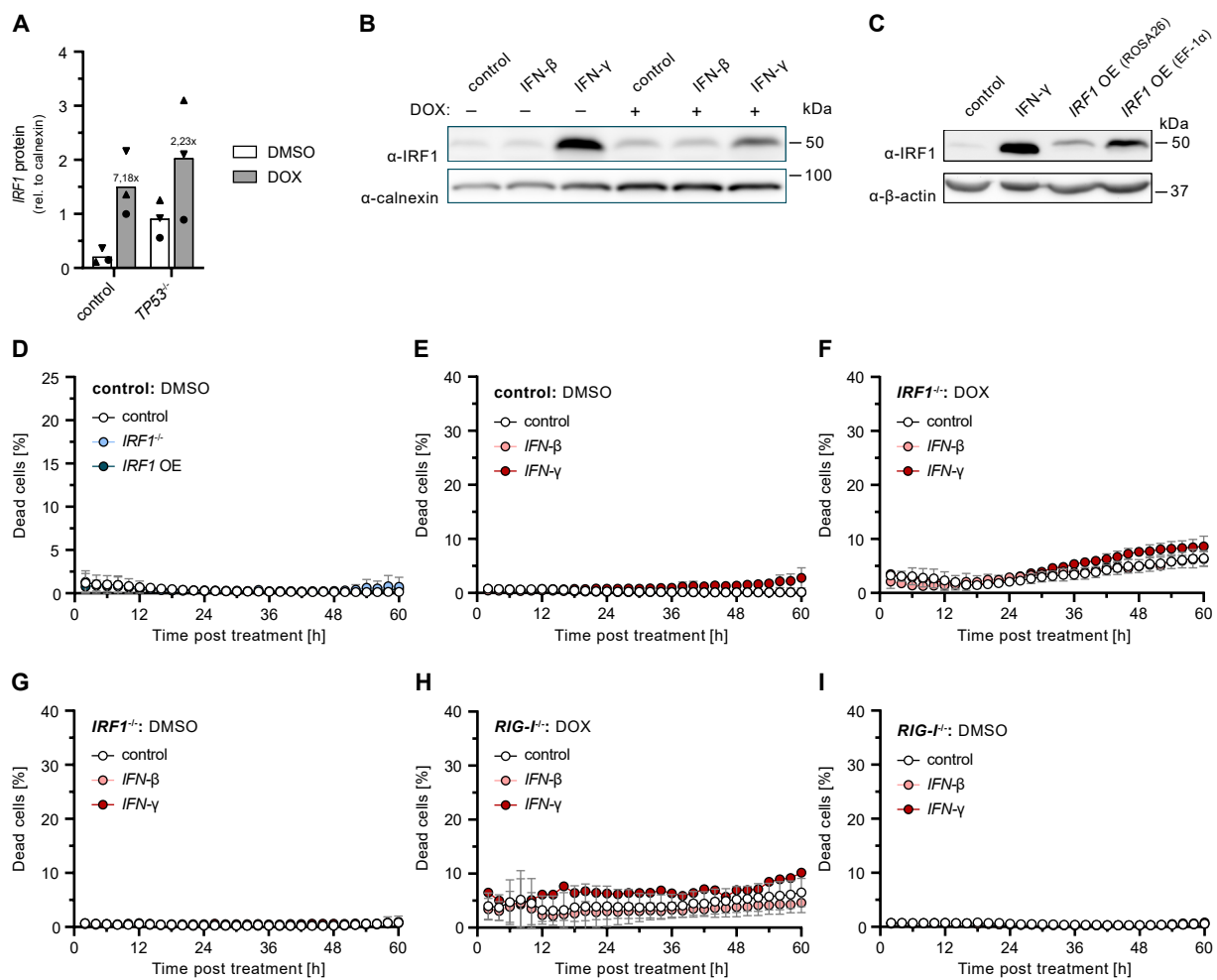

**Fig. S4**

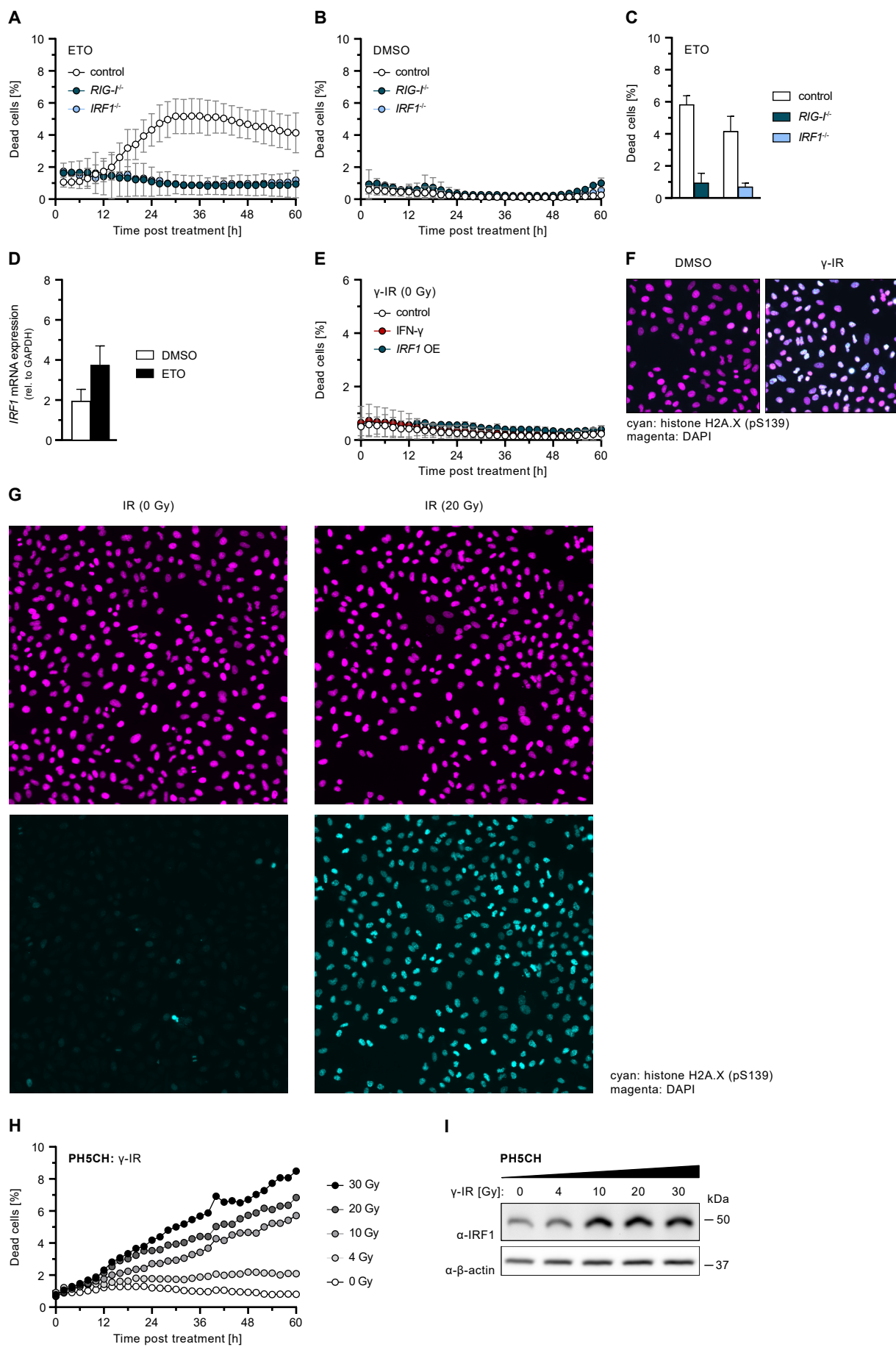

**Fig. S5**

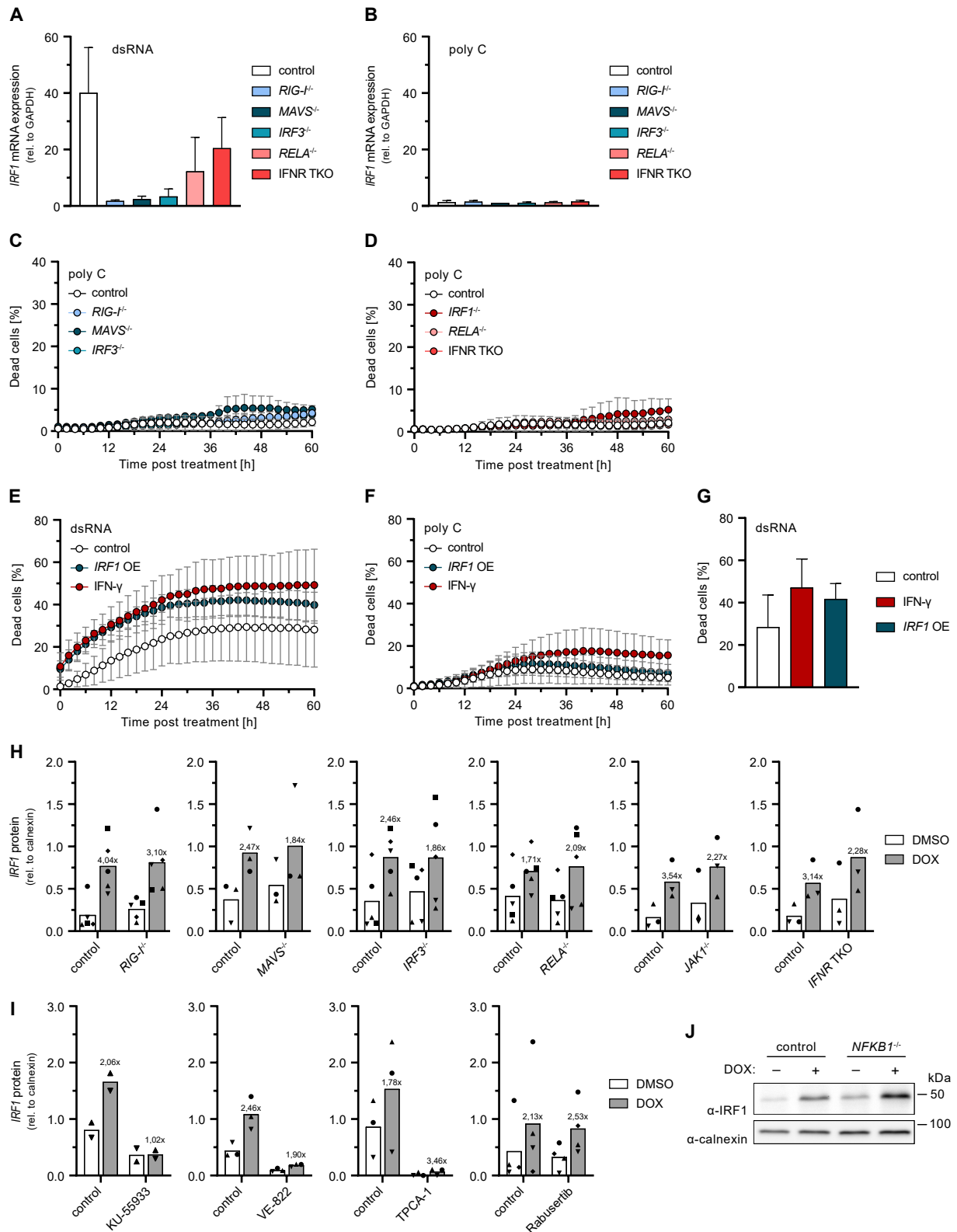

Fig. S6

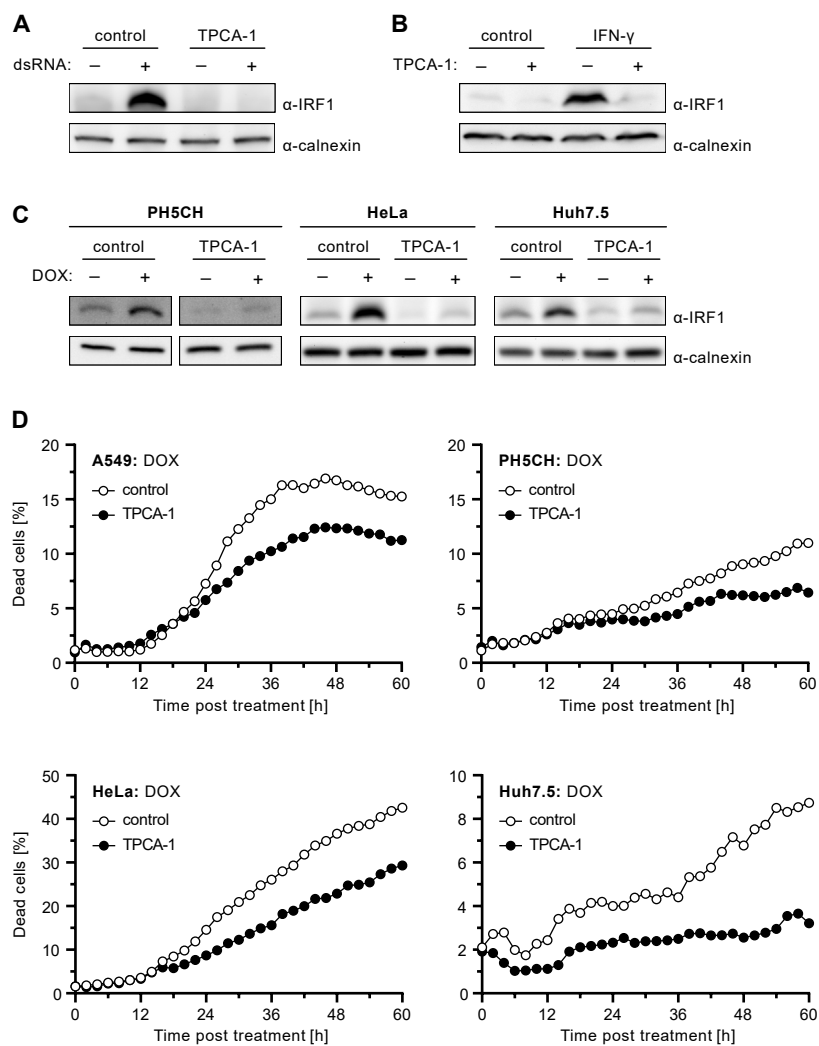

**Fig. S7**

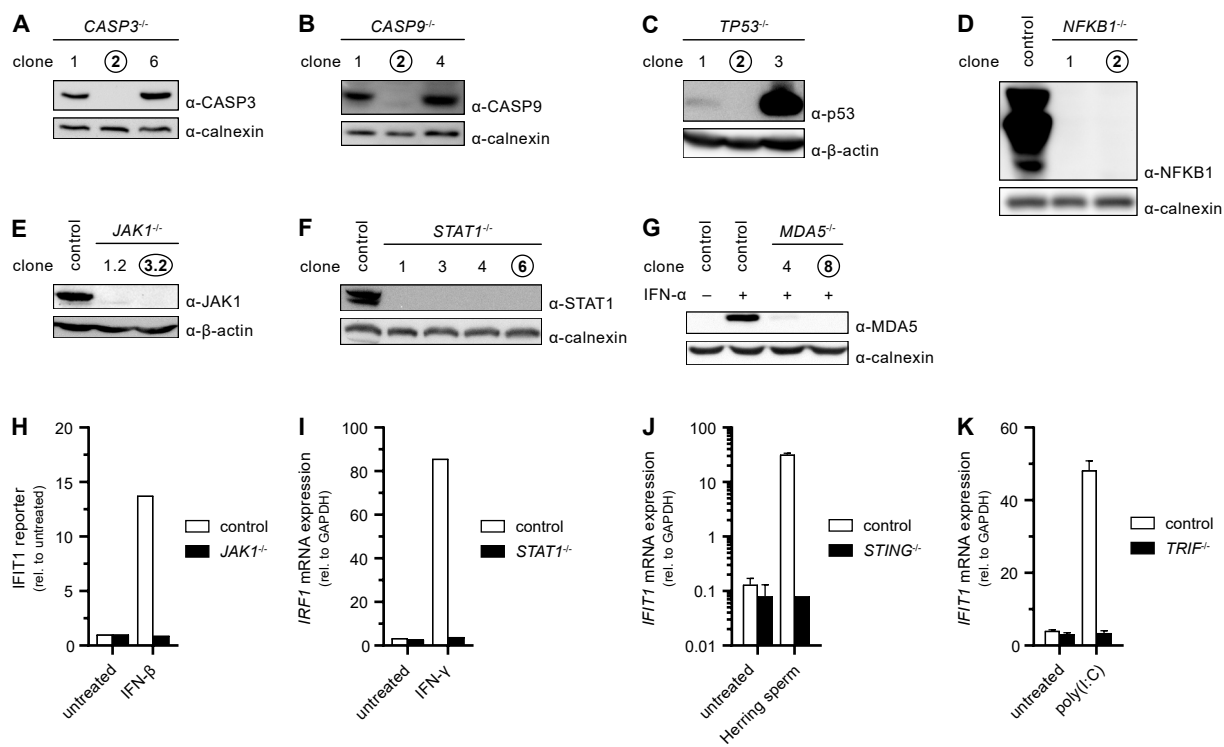

**Fig. S8**

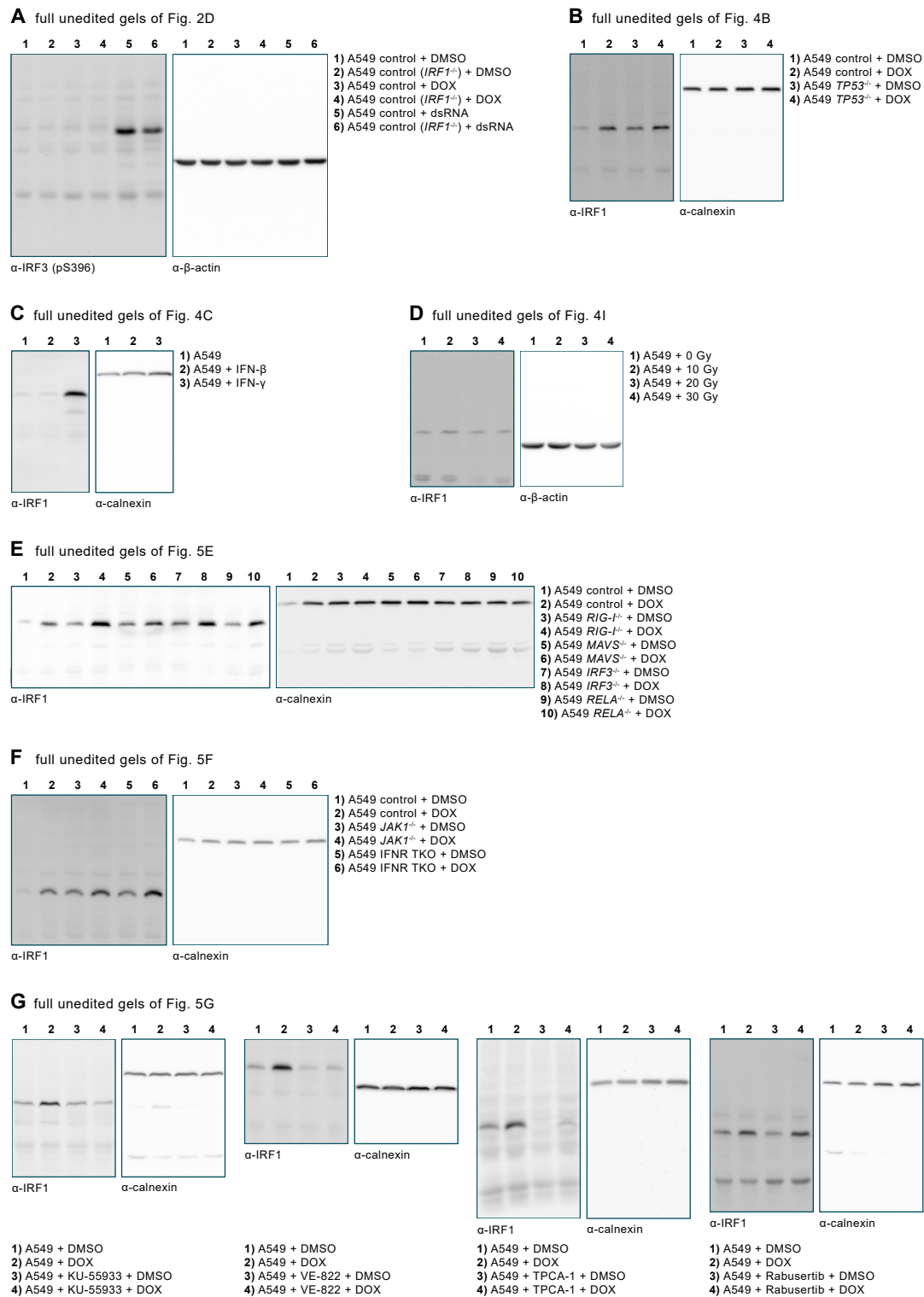

**Fig. S9**

Supplement: Supplementary file 1 — Supplementary Figures [file 41419_2022_4797_MOESM1_ESM.pdf]
