# Supplementary material for: Cooperative effects of RIG-I-like receptor signaling and IRF1 on DNA damage-induced cell death
Source: Cell Death Dis. 2022 Apr 18;13(4):364. doi: 10.1038/s41419-022-04797-7 (PMC9016077; doi:10.1038/s41419-022-04797-7)
Supplement: Supplementary file 2 — Supplementary figure legends [file 41419_2022_4797_MOESM2_ESM.docx]

**Fig. S1.**

**(A)** Immunofluorescence of phosphorylated histone H2A.X (S139) (cyan) and DAPI-stained nuclei (magenta) in A549 cells post DOX treatment for 4 h. Corresponds to Fig. 1C but with separate channels and wider field of view. **(B-D)** Percentage of dead A549 cells with functional KO of the indicated genes relative to total cells counted over time post control treatment with DMSO. Corresponds to Fig. 1E-H.

**Fig. S2.**

**(A, B)** Percentage of dead A549 cells with functional KO of the indicated genes relative to total cells counted over time post control treatment with DMSO. Corresponds to Fig. 2A-C.

**Fig. S3.**

**(A-D)** Percentage of dead A549 cells with functional KO or OE of the indicated genes relative to total cells counted over time post control treatment with DMSO. Corresponds to Fig. 3A-F. **(E)** Percentage of dead A549 cells with functional KO of STAT1 relative to total cells counted over time post treatment with 1 µM DOX.

**Fig. S4.**

**(A)** Levels of IRF1 in A549 control or TP53^-/-^ cells treated with 1µM DOX or DMSO for 10 h were determined by western blot. Quantification of all three biologically independent repetitions are shown; symbols code for experiments. Corresponds to Fig. 4B. **(B)** IRF1 protein levels of A549 cells treated with 1µM DOX or DMSO and stimulated with IFN-β or -γ or mock stimulation. Corresponds to Fig. 4C. **(C)**IRF1 protein levels in mock or IFN-γ treated A549 control cells and A549 IRF1 overexpressing (OE) cells were determined by western blot. Two different promoters are shown (ROSA26, EF-1α), further IRF1 OE experiments are done using the EF-1α construct. **(D, E, G)** Percentage of dead A549 cells with functional KO or OE of IRF1 (D) or stimulated with IFN-β or -γ relative to total cells counted over time post control treatment with DMSO. Corresponds to Fig. 4D, E, G. **(F)** Percentage of dead A549 IRF1^-/-^ cells stimulated with IFN-β or -γ relative to total cells counted over time post treatment with 1 µM DOX. **(H, I)** Percentage of dead A549 RIG-I^-/-^ cells stimulated with IFN-β or -γ relative to total cells counted over time post treatment with 1 µM DOX (H) or DMSO (I).

**Fig. S5.**

**(A-C)** Percentage of dead A549 cells with functional KO of the indicated genes relative to total cells counted over time (A, B) or 36 h (C) post ETO (A, C) or DMSO (B) treatment. **(D)** IRF1 mRNA levels of A549 cells treated with 25 µM ETO for 10 h, measured by qRT-PCR relative to GAPDH transcripts. **(E)** Percentage of dead A549 cells treated with IFN-γ or A549 IRF1 OE cells counted over time in the absence of γ-IR. Corresponds to Fig. 4F. **(F, G)** Immunofluorescence of phosphorylated histone H2A.X (S139) (cyan) and DAPI-stained nuclei (magenta) in A549 cells 1 h post γ-IR. (G) shows wider field of view and separate channels. **(H)** Percentage of dead PH5CH cells relative to total cells counted over time post γ-IR. Data represent one biological experiment with 12 technical replicates. **(I)** PH5CH cells were treated with γ-IR at different doses. After 8 h IRF1 protein levels were determined by western blot. (**A-E**) Data shown represent the results of at least three biologically independent experiments.

**Fig. S6.**

**(A, B)** IRF1 transcript levels in A549 cells with functional KO of the indicated genes upon transfection of 5’ppp-dsRNA (A) or non-stimulatory poly(C) (B) determined by qRT-PCR relative to GAPDH. Corresponds to Fig. 5A. **(C, D)** Percentage of dead A549 cells with functional KO of the indicated genes relative to total cells counted over time upon transfection of poly(C). Corresponds to Fig. 5B-D. **(E-G)** Percentage of dead A549 cells with *IRF1* OE or post IFN-γ pre-stimulation relative to total cells counted over time (E, F) or 36 h (G) post transfection of 5’ppp-dsRNA (E, G) or poly(C) (F). **(H, I)** A549 cells with functional KO of the indicated genes or administration of the indicated inhibitors were treated with 2 µM DOX or DMSO for 6 h. Levels of IRF1 were determined by western blot, and quantified relative to the corresponding calnexin levels. Symbols code for corresponding experiments, bars represent the means. Corresponds to Fig. 5E-G. **(J)** Western blot of IRF1 levels in A549 control or NFKB1^/-^ cells upon treatment with 1 µM DOX or DMSO. (**A-I**) Data shown represent the results of at least three biologically independent experiments.

**Fig. S7.**

**(A, B)** A549 cells were treated with TPCA-1, and stimulated with 1.5 ng/ml dsRNA, poly(C), or 200 IU/ml IFN-γ for 6 h. Levels of IRF1 were determined by western blot. **(C)** PH5CH, HeLa, and Huh7.5 cells treated with TPCA-1, and 2 µM DOX or DMSO for 6 h. Levels of IRF1 were determined by western blot. **(D)**  Percentage of dead A549, PH5CH, HeLa and Huh7.5 cells relative to total cells counted over time post administration of TPCA-1 and DOX treatment; data represent one biological experiment with 12 technical replicates.

**Fig. S8.**

(**A-G**) Functional KO of the indicated genes in A549 cells was validated by determination of respective proteins by western blot. For *MDA5*, gene expression was increased by pre-stimulation with 200 IU/ml IFN-α (**G**). Circles indicate selected clones with validated KO. (**H**) Gene KO was functionally tested by *Firefly* luciferase-based IFIT1 reporter assay post treatment with IFN-β. Firefly was normalized to Renilla luciferase signal, and values were plotted relative to untreated controls. (**I-K**) Functional KO of the indicated genes was tested by determination of *IRF1* (**I**) *IFIT1* (**J, K**) mRNA transcripts post treatment with IFN- γ (**I**), or Herring sperm DNA and poly(I:C) (**J, K**) by qRT-PCR.

**Fig. S9.**

**(A-G)** Uncropped and unedited images of western blots shown in the indicated main figures.
